# Editorial: Necrotrophic Fungal Plant Pathogens

**DOI:** 10.3389/fpls.2022.839674

**Published:** 2022-02-11

**Authors:** Antonieta De Cal, Paloma Melgarejo, Maria Del Mar Jimenez-Gasco

**Affiliations:** ^1^Plant Pathology Department, Instituto Nacional de Investigación y Tecnología Agroalimentaria (INIA), Madrid, Spain; ^2^Ministerios de Agricultura, Pesca y Alimentación (MAPA), Madrid, Spain; ^3^Department of Plant Pathology and Environmental Microbiology, The Pennsylvania State University (PSU), State College, PA, United States

**Keywords:** toxins, necrotrophs, virulence effectors, lytic enzymes, apoptosis, elicitors

Fungal pathogens are a heterogeneous group of organisms which differ in many important traits such as mode of nutrition, type of reproduction, and dispersal mechanisms. Traditional classification of fungal pathogens has relied on their mode of nutrition to classify them into three broad categories viz. biotrophs, necrotrophs, and hemibiotrophs. Biotrophs derive nutrients and energy from living cells, while necrotrophs derive their energy from dead or dying cells. Hemibiotrophs initially invade live cells prior to transitioning to a necrotrophic lifestyle to obtain nutrients from killing the host cells.

We have attempted, in this Research Topic, to take a holistic view of Necrotrophic Fungal Plant Pathogens which provides insights into the aspects that need to be considered in their life cycle. This topic involves integrating knowledge about key aspects of the pathogenesis process, from understanding the demarcation between different trophic lifestyles, to screening for potential genes that selectively activate either to adapt to colonize and disintegrate the intercellular matrix or to break down cell walls and cell compartments in necrotrophic interactions, or host immune responses.

The Research Topic includes manuscripts on the importance of understanding trophic lifestyles. Improving our understanding of necrotrophic pathogens and providing the basis for identifying the complementary host resistance components have been two important points discussed in the present issue by Rajarammohan and Huang S. et al.

This Topic also includes manuscripts that examine in more detail the selectively activated genes either for adapting to colonize and disintegrate the intercellular matrix or for breaching cell walls and cellular compartments in necrotrophic interactions. Contributions to this topic are related to effect of genes controlling development and pathogenicity (Acosta Morel et al.; Malvestiti et al.; Souibgui et al.; Wu et al.), effectors of plant necrotrophic fungi (Duhan et al.; Shao et al.) and enzymes and transcription factors which regulates the growth and pathogenicity (Cheon et al.; Huang Y. et al.; Zhang et al.).

Other manuscripts address the approaches used for the discovery of PRs (Li et al.), phytoalexin effects on necrotrophic pathogens (Huang et al.; N'Guyen et al.), host adaptation to pathogens (Jangir et al.), defense strategies with endophytic fungi (Shi et al.), and detoxification (Westrick et al.) during plant-pathogen interactions. Mechanisms underlying hormone-dependent trophic divergence have been long recognized (Koley et al.).

In addition, the advent of genome sequencing, proteomes (Rodríguez-Pires et al.), and RNAseq (Liu et al.) approaches have been used beneficially to improve studies about a role in suppressing host responses including host cell death (Shao et al.).

Necrotrophic Fungal Plant Pathogens also include manuscripts related to control of diseases caused by necrotrophic fungi (Backes et al.; Marquez et al.; McCaghey et al.; Ostos et al.; O'Sullivan et al.; Testempasis et al.), or fungal populations (Newman and Derbyshire) and their ambient (Balsells-Llauradó et al.), and host adaptation (Plesken et al.).

We believe that these various components provide up to date relevant and useful scientific and translational applied knowledge which will hopefully be useful to scientists and students who want a holistic view of the state of this research area at the present.

## Author Contributions

AD, PM, and MJ-C contributed to guest editing by promoting and participating in the frontiers peer-review process. All authors contributed to the article and approved the submitted version.

## Conflict of Interest

The authors declare that the research was conducted in the absence of any commercial or financial relationships that could be construed as a potential conflict of interest.

## Publisher's Note

All claims expressed in this article are solely those of the authors and do not necessarily represent those of their affiliated organizations, or those of the publisher, the editors and the reviewers. Any product that may be evaluated in this article, or claim that may be made by its manufacturer, is not guaranteed or endorsed by the publisher.

